# Effects of indole-3-carbinol on steroid hormone profile and tumor progression in a mice model of canine inflammatory mammarycancer

**DOI:** 10.1186/s12885-018-4518-z

**Published:** 2018-06-04

**Authors:** Asunción Martín-Ruiz, Laura Peña, Alfredo González-Gil, Lucía Teresa Díez-Córdova, Sara Cáceres, Juan Carlos Illera

**Affiliations:** 1 Department of Animal Physiology, Veterinary Medicine School, Complutense University of Madrid, Madrid, Spain; 2 Department of Animal Medicine, Surgery and Pathology, Veterinary Medicine School, Complutense University of Madrid, Madrid, Spain

**Keywords:** Indole-3-carbinol (I3C), Canine inflammatory mammary cancer (IMC), Inflammatory breast cancer (IBC), Steroid hormones, Mice model, Xenograft

## Abstract

**Background:**

Indole-3-carbinol, derived from Cruciferous vegetables is an estrogen receptor antagonist considered a preventive agent that is naturally present in diet. There are no previous studies on its effects in human inflammatory breast cancer or canine inflammatory mammary cancer that is the most aggressive type of breast cancer.

**Methods:**

The aim of this study was to analyze the effect of indole-3-carbinol on a SCID mice xenograft model of canine inflammatory mammary cancer, using equivalent human oral dose as a preventive therapy in humans for 3 weeks.

**Results:**

Indole-3-carbinol treatment decreased tumor proliferation and increased apoptosis, although tumor embolization and liver metastasis were observed in some animals. There was a characteristic subpopulation of lipid-rich cells and increased contents of select steroid hormones in tumor homogenates and serum.

**Conclusions:**

Our data reveal for the first time that the ingestion of indole-3-carbinol, as administered, diminishes proliferation and increases apoptosis of tumor cells in an experimental model of inflammatory breast cancer, although this effect could not be enough to avoid the appearance of tumor embolization and metastasis. Future clinical trials will be needed to clarify the usefulness of indole-3-carbinol in this cancer and to understand the molecular mechanisms involved.

## Background

The relationship between cancer and nutrition has been largely studied; epidemiologic studies indicate that consumption of vegetables containing dietary phytochemicals reduces the risk of developing cancer [1]. Dietary phytochemicals are a wide variety of biologically active compounds found in plants that contain anti-tumor and anti-inflammatory properties [2–5]. Many of these substances block tumor growth and inhibit metastasis in animal models [6, 7]. Studies on synergistic effects of different phytochemicals might contribute to establish potential chemopreventive strategies [8, 9]. Indole-3-carbinol (I3C), a natural phytochemical found in cruciferous vegetables (i.e. broccoli, cabbage or cauliflower), is considered a potential anticancer agent that prevents the development of certain types of tumors by activating tumor suppressor genes, genes involved in apoptosis and detoxification [2, 10–14]. Some in vitro and in vivo studies in breast cancer [15–20] and other neoplasia types such as colorectal cancer, prostate cancer, ovarian cancer, cervix carcinoma and hepatocarcinoma indicated that I3C suppresses cell proliferation and induces apoptosis [17, 21–25].

Several in vitro studies with breast cancer cells showed that I3C acts by blocking estrogen receptors among other mechanisms [10, 17, 26]. Thus, some authors showed that I3C might be useful as supplement of tamoxifen preventing or treating estrogen-dependent tumors [27, 28]. There are no data on the effect of I3C in human inflammatory breast cancer (IBC) or in spontaneous and experimental canine inflammatory mammary cancer (IMC). Inflammatory breast cancer and IMC are considered as a special clinic-pathological entity and the most malignant type of breast cancer both in humans and dogs, with a fulminant clinical course and an extremely poor survival rate [29–34]. Inflammatory breast cancer has been proposed as a natural model to study the human disease [29, 35, 36]. Our group has published the establishment and validation of a xenograft model of canine IMC in mice [37]. Spontaneous and experimental canine IMC have been associated with high levels of steroid hormones in tumor homogenates, suggesting a potential autocrine/paracrine secretion [37–41].

The aim of this study was to analyze the hypothetical antitumor effect of I3C administration on a SCID mouse xenograft model of canine inflammatory mammary cancer using an equivalent dose to that used as a preventive anti-cancer agent in humans.

## Methods

### Animals and xenograft establishment

The protocol was approved by the Committee of the Universidad Complutense of Madrid, and Animal Protection Area of the Community of Madrid, Spain (Ref. Protocol Number: PROEX 31/15). Twenty-four non-ovariectomized female SCID mice (BALB/cJHan®Hsd-*Prkdc*^scid^, Harlan Laboratories Models, S. L.), 6–8 weeks of age and weighing between 20 and 22 g were used. The animals were housed in a flexible-film isolator (Racks IVC de Allentown Inc. Panlab Harvard Apparatus) in cages (2–3 animals per cage), each measuring 330 cm^2^ × 12 cm, in a room with controlled environmental conditions (20 °C to 22 °C; 50 to 55% relative humidity; 10 to 15 air changes per hour; and a 12:12 h light: dark cycle). Pre-sterilized food and water were provided ad libitum. All experimental procedures were performed between 11:30 a.m. and 12:30 a.m. The method of euthanasia used to sacrifice the mice was isoflurane flow rate adjusted to 5% until one minute after breathing stops. Then, cervical dislocation was applied as method of confirmation of euthanasia.

The xenograft was directly established from a 9-year-old female dog with a spontaneous inflammatory mammary carcinoma, following a protocol previously established [37] as follows: Fragments (3 mm × 2 mm of diameter) from canine primary mammary tumor obtained at necropsy were immediately placed in MEM (Minimum Essential Medium) with Earle’s Salts, L-Glutamine and Penicillin/Streptomycin [100×] (*PAA Cell Culture Company*, *BioPath Stores*, *Cambridge*) before they were subcutaneously implanted into the ventral side of 3 female SCID mice. The mice were previously anesthetized with isoflurane (*IsoVet 1000 mg/g, B Braun VetCare SA, Barcelona, Spain*) at 4% for induction and at 1.5% for maintaining anesthesia. Isoflurane was supplied in a fresh gas flow rate of 0.5 l of oxygen/minute. The mice were monitored for tumor growth and, whenever palpable, the xenograft tumor volumes were measured twice weekly with a caliper-like instrument throughout the experiment, and the tumor size was estimated using the following formula: *(L × W*^*2*^*)/2*, where *W* = width and *L* = length. When the implanted tumors reached approximately 1.0 cm^3^, they were successively transplanted into three SCID mice (second passage) and from each of these animals to three new animals (third passage, *n* = 30), in order to verify that the xenograft model was stable and that these tumors did not present histopathological modifications between the consecutive passages. The engraftment efficiency of the second and third passages was 90% (*n* = 24).

### Treatment groups

Animals of third passage were randomly assigned to an I3C treatment/control group (*n* = 12/12). All mice from the two groups were treated by oral gavage: the control group, administered with 200 μl of distilled water/polyethylene glycol (*Panreac Quim. S.A, Barcelona, Spain*) (6:4 ratio, *n* = 12/24) and the I3C group (administered with 200 μl of distilled water/polyethylene glycol containing 150 mg I3C/kg/day) (Indole-3-carbinol, *Sigma-Aldrich Co., Madrid, Spain*), (*n* = 12/24). The dose was chosen from previous studies [42, 43]. Mice were given the doses through an oral feeding tube (18G x ½”) for 3 weeks (the first dose was given 7 days after the xenografts, when tumors were palpable), following a dosage of 5 consecutive days of administration and 2 days of rest. The operator was blinded to the treatment group assignment of each animal at all times.

### Histopathology and immunohistochemistry

Fragments from the treated tumors were fixed in neutral formalin and then embedded in paraffin for tumor histopathology. The samples were histologically diagnosed on HE-stained sections following the histological classification of canine mammary tumors [44]. Histology and immunohistochemistry of samples were evaluated by an experienced veterinary pathologist.

Immunohistochemistry for Ki-67, caspase-3 and estrogen receptor (ER) was performed on deparaffinised sections and using the streptavidin–biotin–complex peroxidase method or detection kits. High-temperature antigen retrieval with 10 mM citrate buffer pH = 6.0 was performed. Table 1 shows the primary antibodies and developing systems used. In the case of caspase-3, the slides were incubated with streptavidin-HRP conjugated anti-caspase-3 antibody (*TermoFisher Scientific* ref. 43–4323, dilution 1/4000) for 30 min at room temperature (RT) and developed with a chromogen solution containing 3, 3′-diaminobenzidine tetrachloride (DAB). Thereafter, the slides were counterstained with hematoxylin. Washes and dilutions were made in Tris-buffered saline (pH = 7.4) for each marker; corresponding positive and negative control slides were performed*.*

**Table 1:** Primary and secondary antibodies/kits used for immunohistochemistry

Primary Antibody	Type	Source	Incubation	Secondary Antibody/kit	Source	Incubation
Ki-67 clon MIB-1 (Ref. M7240)	Mab	Dako 1:75	60 min, RT	EnVision+SystemHRP (DAB) (Ref. K4007)	Dako kit	40 min, RT
ERα clon 1D5 (Ref. M7047)	Mab	Dako 1:15	Overnight 4 ° C	EnVision + SystemHRP (DAB) (Ref. K4007)	Dako kit	40 min, RT
Caspase-3 (Ref. AF835)	Pab	R&D Systems 1:1200	Overnight 4 ° C	Swine anti-rabbit biotinylated (Ref. E0353)	Dako 1:200	30 min, RT

*Mab* mouse monoclonal antibody, *Pab* rabbit polyclonal antibody

Tumor proliferation index (PI) was determined by counting Ki-67 positive and negative nuclei in 8–10 selected High Power Fields (HPF) with the highest percentage of labeling (minimum 1000 cells). Every immunostained nucleus was considered positive regardless of the intensity of stain. The PI or proportion of positive neoplastic cells in each sample was calculated as previously described [44].

Caspase-3 immunostaining was performed to analyze the presence of apoptotic bodies in the xenograft tissue and it was semi-quantitatively assessed by the intensity of immunoexpression, which was evaluated as low (+), moderate (++), or intense (+++), by the percentage of positive cells (apoptotic index).

Finally, the expression of ER was considered positive when more than 10% of the cells within the tumors were positive independently of the intensity of the staining [29].

### Steroid hormone concentrations in serum samples and tumor homogenates

At the time of necropsy of the control and experimental SCID mice, 1 ml of blood was obtained by cardiac puncture using a 25G needle from each mouse. Blood samples were collected in special tubes (microtube Serum-Gel Clotting Activator, *Sarstedt*, *Nümbrecht*, *Germany*) and spun down in a refrigerated centrifuge (*Hettich Zentrifugen Universal 320 R*, *Germany*) at 4 °C and a speed of 1200 x g (RCF) for 20 min. Serum was harvested and stored at − 20 °C until assayed for the presence of hormones. Frozen tissue specimens of approximately 0.1–0.5 g from individual mice were homogenized in 5 ml of PBS (pH = 7.2) and the homogenate centrifuged at 1200 x g, for 20 min at 4 °C. The supernatants were harvested and stored at − 80 °C until hormone assays [45].

Levels of estrone sulphate (E1SO_4_), estradiol (E2), androstenedione (A4), testosterone (T), dehydroepiandrosterone (DHEA) and progesterone (P4) in tumor homogenates and serum samples were assayed by amplified Enzyme-Immunoassay (EIA) previously validated in our laboratory [45].

### Statistical analysis

Statistical analyses were performed by the IBM SPSS Statistics, 19.0 for Windows (Chicago, IL, USA). The relationship between continuous variables (plasma and tumoral steroid hormone levels, Ki-67 and caspase index) and categorical variables (pathological parameters such as ulceration, sebaceous hyperplasia, dermal emboli, tumor emboli, degenerated emboli, thrombosis, lipid-rich subpopulation, distant metastases, liver metastasis and estrogen receptor alpha) was established using the analysis of variance (ANOVA) followed by appropriate post hoc tests for similar variances (Duncan Test) or different ones (Games-Howell test). The relationship between continuous variables was assessed using the Pearson correlation. The association between categorical variables was analyzed using Pearson’s chi-squared test (χ^2^). Differences were considered significant at *p-value* < 0.05.

## Results

### Macroscopic growth and metastases

A stable serial transplantable xenograft was successfully established with a constant and rapid growth in all mice. At the end of the experiment, in 4 weeks, the tumors had an approximate size of 0.9–1.2 cm^3^. The size and weight of the xenograft tumors in I3C treated and untreated mice (controls) did not show significant differences (*p* > 0.05).

Immediately after sacrifice, complete necropsy was performed in each case. Common target organs for IMC such as bone, lung, liver, brain or distant lymph nodes were extensively examined. At necropsy, one xenotrasplanted control mouse presented pulmonary and mesenteric metastases (1/12, 8.3% of the control group) and one out of 12 (8.3%) I3C treated xenografts had pulmonary and mesenteric metastases. Four animals in the I3C group showed metastasis in the liver (4/12, 33.3%, *p* = 0.028). All metastases were histologically confirmed.

### Histopathological findings

#### Primary canine IMC and control xenografts

**Fig. 1:**
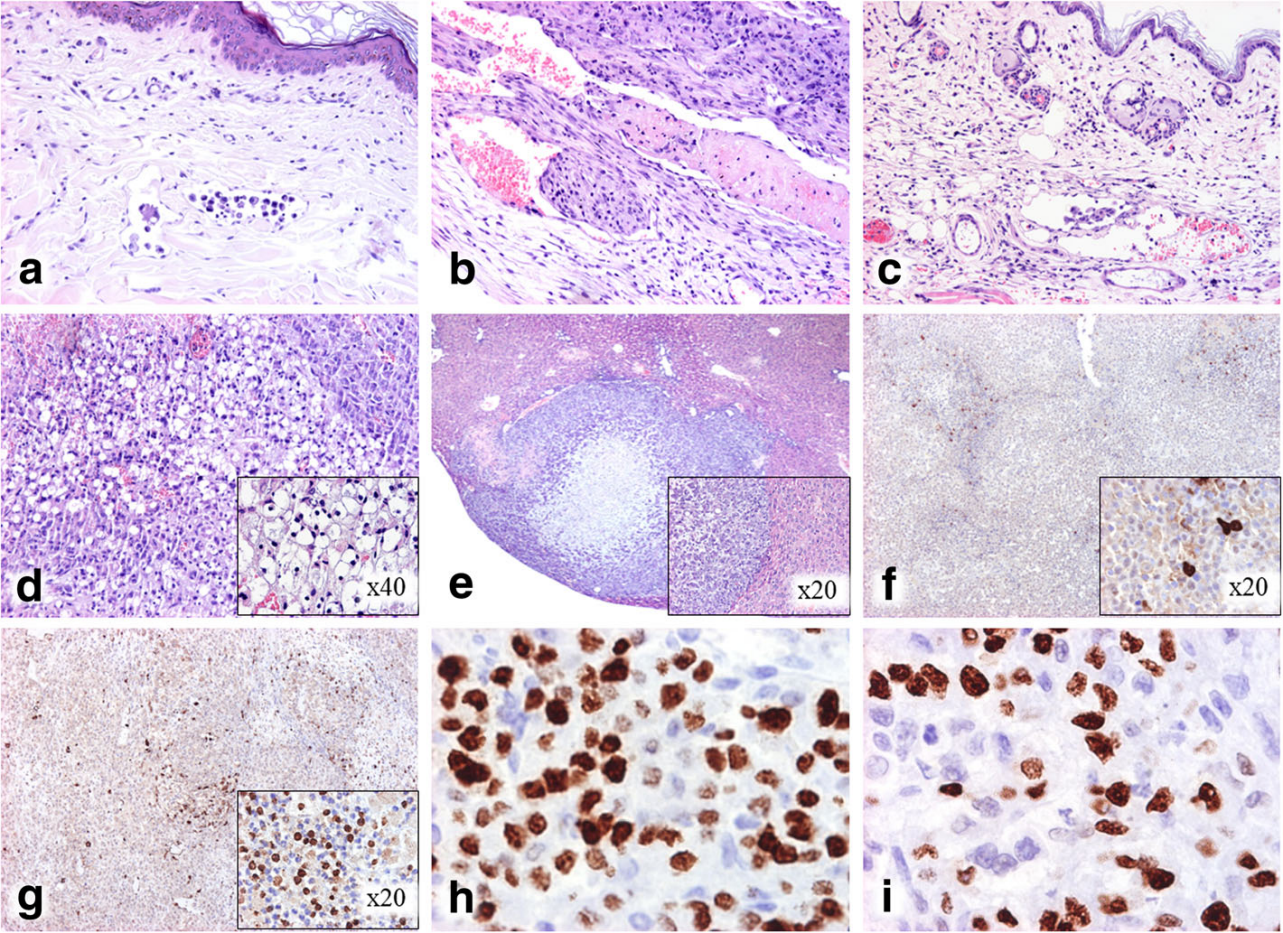
Representative histological features. **a** IMC with neoplastic emboli in superficial dermal lymphatic vessels and infiltration of carcinomatous cells in the female dog origin of the xenografts. **b** IMC with tumor emboli in SCID mouse (BALB/cJHan®Hsd-*Prkdc*^*scid*^) xenograft control group. **c** IMC with tumor emboli in dermis in SCID mouse xenograft I3C group (*p* = 0.012, compared with control group), and non-dermal tumor emboli in I3C mice (*p* = 0.035). **d** IMC with abundant lipid-rich cells in SCID mouse xenograft I3C group (× 20), (× 40) (*p* = 0.001). **e** IMC with liver metastasis in SCID mouse xenograft I3C group (× 2), (× 20) (*p* = 0.028). **f** and **g** IMC showing caspase-3 positive immunolabeling in a low number of cells in SCID mouse xenograft control group versus I3C group where positive immunolabeling in numerous cells (× 4), (× 20) (*p* < 0.001). **h** IMC showing positive Ki-67 immunolabeling in numerous cells in xenograft control group, and **i** lower number of Ki-67 positive cells in the I3C group SCID mouse xenograft (× 20) (*p* < 0.001). Analysis of variance followed by appropriate post hoc tests for similar variances (Duncan Test) or different ones (Games Howell test) was used. *IMC* inflammatory mammary carcinoma, *I3C* indole-3-carbinol

According to the clinical characteristics and the histological invasion of dermal lymphatic vessels by tumor/neoplastic emboli (Fig. 1a), the primary canine mammary tumor was diagnosed as an inflammatory mammary cancer originated by a highly undifferentiated anaplastic/solid mammary carcinoma grade III with scattered lipid-rich cells. Control xenografts reproduced the histological features of the primary canine mammary tumor. Hence, emboli at peripheral lymphatic and blood vessels, as well as inside the tumor were observed (2/12, 16.7%) (Fig. 1b). The control xenografts were highly infiltrative in the dermis and striated muscle and were partially surrounded by a fibro-myxoid tissue. Neoplastic cells displayed a solid pattern together with areas of isolated cells; the stroma was scant. Scattered tumor cells showed a lipid-rich cytoplasm. The neoplastic cells exhibited a high pleomorphism with marked cell and nuclear atypia, and an elevated mitotic index (anaplastic cells). Binucleated and multinucleated cells, as well as atypical mitoses were frequently found. Large intratumor areas of necrosis, dermal lymphangiectasias, and severe edema in the dermis were also present.

#### Histological and immunohistochemical findings in the I3C treated xenografts

Histological and immunohistochemical findings of both experimental groups are depicted in Table 2. Indole-3-carbinol treatment diminished proliferation and increased apoptosis of tumor cells although embolization of neoplastic cells (7/12), liver metastasis and presence of tumor cells showing a lipid-rich cytoplasm (8/12) (Figs. 1c-i) together with hyperplasia of dermal sebaceous glands (10/12) were also observed in some animals. This lipid-rich cell subpopulation frequently presented degeneration (8/12) and necrosis.

**Table 2:** Histopathological and immunohistochemical characteristics in control and indole-3-carbinol (I3C) xenograft groups

	Control xenograft (%)	I3C xenograft (%)	*p-*value
Ulceration	33.3	0.0	***p*** **= 0.028**
Sebaceous hyperplasia	8.3	83.3	***p*** **< 0.001**
Dermal emboli	0.0	41.7	***p*** **= 0.012**
Tumor emboli (non-dermal)	16.7	58.3	***p*** **= 0.035**
Degenerated emboli	0.0	8.3	*p* > 0.05
Thrombosis	33.3	0.0	***p*** **= 0.036**
Lipid-rich subpopulation	0.0	66.7	***p*** **= 0.001**
Distant metastases (any location)	8.3	33.3	*p* > 0.05
Liver metastases	0.0	33.3	***p*** **= 0.028**
	Control xenograft (%, mean ± standard deviation)	I3C xenograft (%, mean ± standard deviation)	*p-*value
Ki-67 index	62.2 ± 4.55	54.5 ± 3.79	***p*** **< 0.001**
Caspase index	6.9 ± 1.35	27.5 ± 2.33	***p*** **< 0.001**

*P* values in boldface are significant. These values are *p* < 0.05

Primary canine tumor and all xenografts samples were negative for ER immunostaining. The positive control was canine uterus.

#### Determination of steroid hormones

The content of steroid hormones in serum and tumor homogenates of both control and I3C xenograft groups are shown in Table 3. Significantly higher levels of estrone sulphate (*p* = 0.009), estradiol (*p* < 0.001) and androstenedione (*p* = 0.049) and lower level of testosterone (*p* = 0.048) in I3C xenograft homogenates compared with the control group were found. Serum progesterone (*p* = 0.003) and testosterone levels (*p* = 0.022) were also higher in I3C group.

**Table 3:** Serum (S) (ng/ml) and tumor homogenate (T) (ng/g) steroid hormone concentrations in control and indole-3-carbinol (I3C) groups after 3 weeks of treatment

		Control xenograft	I3C xenograft
Estrone sulphate (E1SO4)	S	0.12 ± 0.03 ^a,1^	0.24 ± 0.05 ^a,1^
	T	13.71 ± 2.42 ^a,2^	27.09 ± 3.09 ^b,2^
Estradiol (E2)	S	11.98 ± 0.82 ^a,1^	12.62 ± 1.01 ^a,1^
	T	7.44 ± 0.60 ^a,2^	13.89 ± 0.99 ^b,1^
Androstenedione (A4)	S	5.96 ± 0.77 ^a,1^	6.10 ± 1.28 ^a,1^
	T	5.16 ± 0.99 ^a,1^	20.46 ± 5.17 ^b,2^
Testosterone (T)	S	1.20 ± 0.09 ^a,1^	1.76 ± 0.25 ^b,1^
	T	12.16 ± 3.07 ^a,2^	4.02 ± 1.21 ^b,1^
Dehydroepiandrosterone (DHEA)	S	2.96 ± 0.16 ^a,1^	3.06 ± 0.37 ^a,2^
	T	2.32 ± 0.11 ^a,1^	2.86 ± 0.63 ^a,2^
Progesterone (P4)	S	0.69 ± 0.07 ^a,1^	1.15 ± 0.10 ^b,1^
	T	5.37 ± 1.04 ^a,2^	7.75 ± 1.98 ^a,2^

All values expressed as mean ± standard error. Different letters denote significant differences (*p* < 0.05) among groups. Different numbers denote significant differences (*p* < 0.05) between serum samples and tumor tissue homogenates, in the same treatment group

## Discussion

Several experimental studies have shown that I3C possesses preventive anti-cancer [15, 17, 22] and disrupting estrogen signalling properties [10, 46–48]. Indole-3-carbinol is considered a potential agent in the prevention and treatment of hormone-dependent breast tumors [2, 21]. Thus, it is freely available in the stores and supermarkets as a dietary phytochemical (non as an approved pharmaceutical drug) for preventing cancer, diminishing premenstrual syndrome and perimenopause-related disturbances. Nevertheless, the effect of I3C on patients with neoplasms, especially breast cancer is poorly documented [20, 49]. To the best of our knowledge, this is the first study regarding the effects of I3C treatment on experimental or spontaneous IMC. Both IBC and canine IMC are considered a special and very aggressive type of breast cancer due to its particular/unique biological, molecular, pathological, genetic and clinical signature features [30, 32–34]. In the present study, I3C treatment on a xenograft model of canine IMC reduced tumor growth and increased apoptosis, although metastasis and alterations in the peripheral levels of steroid hormones were also observed in some animals.

Tumor proliferation index has been associated with poor prognosis in human [49] and canine mammary cancer [50]. Our xenografts from the I3C group had lower proliferation index compared with control group, indicating a loss of proliferative capacity after I3C administration. In accordance with our results, several reports using breast cancer cell lines [3, 18, 19, 51, 52] and human breast cancer cell-derived tumor xenografts, have reported an antiproliferative effect of I3C [20, 49]. The antiproliferative property of I3C has been associated to changes in cell signaling, specially disrupting estrogen responsiveness [19, 22, 47, 48]. Indole-3-carbinol could have also a role in the induction of specific carcinogen detoxifying enzymes, such as CYP1A [3, 17, 53], and in cell adhesion, dissemination and invasion of human breast cancer cells [2].

In the present study, the lack of ulceration and increased apoptosis in treated I3C tumors is in accordance with a reduction of tumor growth and with other previous in vitro and in vivo studies in human breast cancer [22, 51, 53, 54], probably through pro-apoptotic and anti-proliferative mechanisms [12, 19, 55, 56]. In addition, it has been reported that I3C promotes apoptosis of breast cancer cells by activating caspase-3 and -9, among other mechanisms [4, 22, 54]. Accordingly, our study showed a high expression of caspase-3 in I3C treated xenografts. Both IBC and IMC are typically angiogenic, lymphangiogenic and lymphangiocentric, being the presence of massive neoplastic emboli in dermal vessels, their main histological feature [29, 35, 57, 58]. The presence of dermal neoplastic emboli in experimental mice IBC/IMC xenotransplant models has been documented but it is not seen in all the samples [37, 57–61]. It is known that the presence of emboli within the dermal lymphatic vessels in IMC, contributes to the rapid development of metastasis [62–64], and is responsible for the morbidity of this disease. In spite of the antiproliferative and apoptosic effect, emboli in dermal lymphatic vessels and liver metastasis were observed in some animals. Therefore, the effect of I3C could not be enough to avoid the appearance of tumor embolization and metastasis.

The appearance of metastases indicates that this biomodel is appropriate for the study of metastatic tumors in general and inflammatory mammary cancer in particular, since both IBC and IMC are very aggressive tumors with an extremely high metastatic potential. The capacity of I3C to inhibit cell adhesion, migration and invasion of non-inflammatory ER- breast cancer cell has been previously reported [2]. However, in this study, metastases were observed in some animals. A potential limitation of the current study is the scarce number of animals used. More studies should be performed to evaluate the capacity of I3C to inhibit the appearance of metastasis in this cancer.

The presence of thrombosis and some necrotic/degenerated neoplastic cells inside the emboli has been previously documented in IMC xenografts and suggests a partial destruction of metastatic cells, reducing the possibility of establishing distant metastasis [37]. In our study, only the control group showed thrombosis, which could suggest a reduced defense mechanism in I3C treated IMC xenografts. The possible host defense mechanisms in these xenografts remain to be elucidated.

The effect of I3C treatment on estrogens metabolism has been previously indicated [10, 27, 28, 53, 65]; nevertheless, to the best of our knowledge, this is the first time that the possible effects of this indole on other steroid hormones and general steroidogenesis have been studied. According to previous studies, the alteration of estrogen metabolism by I3C can be mediated by cytochrome P450 [66, 67] or the activation of aryl hydrocarbon receptor (AhR)-mediated pathways [68–70].

Histopathological evidence of lipid droplets in neoplastic cells has been described in spontaneous and experimental canine IMC, and it has been shown that could be due to steroid secretion [29, 37, 40]. The frequent presence of scattered lipid-rich cells in our murine model has been previously described in another IMC xenograft model [37]. In the present study I3C treatment increased significantly the number of intracellular lipid droplets that appeared forming large areas of lipid-rich cells, which were not present in the control group tumors. According to previous studies, these lipid-rich cells could contain large amounts of steroids that could be locally secreted [37, 38, 40]. The steroid secretion by IBC and IMC cell lines has been recently indicated [71]. The presence of large areas containing a lipid-rich cell subpopulation is in accordance with previous observations and explains the high amounts of steroids found in tumor homogenates of I3C treated xenografts of the present study. According to our results, several steroid hormones (estradiol, estrone sulphate and androstenedione) were significantly increased in I3C treated tumor homogenates while testosterone was diminished. Interestingly, testosterone was significantly increased in the sera of I3C treated mice. Further studies should elucidate if these changes in steroid hormone content are related to a major production of estrogens in situ (via aromatase or sulphatase enzymes).

The mechanisms causing hormonal variations after I3C administration are uncertain. The documented anti-estrogen effect of I3C through ER should be questioned in this particular case, since the model is ERα negative [3, 28, 46, 65]. As pointed out before, I3C can alter the estrogen metabolism through other ER-independent pathways [15, 27, 47, 53]. The role of other receptors such as ERβ should be considered and elucidated in future studies. The relevance of ERβ in the pathogenesis of IMC has been suggested previously [39]. Our study suggests that both E1SO_4_ and E2 can be locally synthesized, especially after I3C administration. Following the aromatase or sulphatase pathways, androgens (especially testosterone that is diminished in tissue) could be transformed in high tissue E1SO_4_ and E2 concentrations. Estrone sulphate could be a reservoir for the synthesis of active estrogens in the mammary tumors, including the canine IMC [39, 40, 72]. Previous studies indicated that I3C effect on breast cancer cells favors estradiol 2-hydroxylation [46, 73]. Although there are no previous studies on intratumoral E2 levels in I3C treated cells, such the activation of estradiol 2-hydroxylation should induce a low level of E2 [19, 74] but an increase of E2 and E1SO_4_ concentrations in tumor homogenates was observed. This particular finding has to be compared in non-inflammatory breast cancer xenografts to elucidate if the high amounts of E2 found in the present study are due to the particular characteristics of IMC. In addition to our results refer to intratumoral levels, as stated above.

The higher concentration of steroids and longer half-life suggests the formation of biologically active estrogens in tumor tissue [40]. In order to proliferate, it is possible that tumor increased estradiol levels from the E1SO_4_ reservoir. In our results, serum P4 concentrations increased in I3C group, probably due to a lower expression of progesterone receptors by I3C, and consequently, higher serum P4 levels [10, 65, 75]. As occurs with estrogens and progesterone a local synthesis of androgens by IBC and IMC tumor cell lines, has also been confirmed [71]. The role of androgens in breast cancer is not clear. Some studies suggest an antiproliferative effect [76], but others note an increased proliferation in breast cancer cell lines [77, 78]. The decrease of testosterone levels in I3C-treated group could be due to the inhibitory effect of I3C on the androgen receptors transcription [66]. Similar to E2, a higher content of intratumoral A4 in I3C group could be due to an increased local synthesis and to the binding of I3C to androgen receptors, increasing A4 free levels in tissue homogenates. However, intratumoral T concentrations decreased in I3C group when compared with control group, probably by the I3C-associated stimulatory effect on the aromatase enzyme [79] which stimulates T to E2 conversion.

## Conclusions

Our data reveal for the first time that the ingestion of indole-3-carbinol, as administered, diminishes proliferation and increases apoptosis of tumor cells in an experimental model of inflammatory breast cancer, although this effect could not be enough to avoid the appearance of tumor embolization and metastasis. Additionally, stimulated steroid hormones levels and a characteristic lipid-rich cell population were presented. These results could be attributable to the special characteristics of this particular cancer. Future clinical trials and studies will be needed to clarify the usefulness of indole-3-carbinol in this cancer and to understand the molecular mechanisms involved.

## Abbreviations

A4: Androstenedione
AhR: Aryl hydrocarbon receptor
DAB: 3, 3′-diaminobenzidine tetrachloride
DHEA: Dehydroepiandrosterone
E1SO_4_: Estrone sulphate
E2: Estradiol
EIA: Enzyme-Immunoassay
ER: Estrogen receptor
HPF: High Power Fields
I3C: Indole-3-carbinol
IBC: Inflammatory breast cancer
IMC: Canine inflammatory mammary cancer
MEM: Minimum Essential Medium
P4: Progesterone
PI: Proliferation index
RCF: Relative Centrifugal Force or G-force
RT: Room Temperature
T: Testosterone
